# Methodological advancement in deriving primary productivity and ecosystem respiration fluxes across different biomes

**DOI:** 10.1016/j.mex.2024.102773

**Published:** 2024-05-21

**Authors:** Aparnna Ravi, Dhanyalekshmi Pillai, Vishnu Thilakan, Thara Anna Mathew

**Affiliations:** a Indian Institute of Science Education and Research Bhopal (IISERB), India; b Max Planck Partner Group at IISERB, Bhopal, India

**Keywords:** Solar induced fluorescence, TROPOMI, Gross primary productivity, Ecosystem respiration, Refinement of LUE-based vegetation model

## Abstract

In this paper, we introduce a methodology that can improve the estimations of Gross Primary Productivity (GPP) and ecosystem Respiration (R_eco_) processes at a regional scale. This method is based on a satellite data-driven approach which is suitable for regions like India where there exists a serious shortage of ground-based observations of biospheric carbon fluxes (e.g., Eddy Covariance (EC) flux measurements). We relied on the Moderate Resolution Imaging Spectroradiometer (MODIS) reflectance for capturing vegetation dynamics in the Light-Use Efficiency (LUE)-based vegetation model. Further, we utilised recently available satellite-based Solar-Induced Fluorescence (SIF) and other variables such as Soil Moisture (SM) and Soil Temperature (ST) to refine the predictions of GPP and R_eco_. The methodology involves establishing a relationship between SIF and GPP for different vegetation classes over India. The SIF-GPP relationship established across the biomes was then used to correct the GPP fluxes simulated by the LUE-based model. Similarly, the ecosystem respiration estimations by the model have undergone refinement by incorporating ST and SM information. This innovative method shows remarkable potential to improve biospheric CO_2_ uptake and release, especially for in situ data-constrained regions like India.

• SIF-based information is introduced to a light-use efficiency-based vegetation model.

• SIF-GPP relationship is established for major biomes across India.

• SM and ST information is incorporated into the R_eco_ simulations in the model.

## Graphical abstract

**Figure:**
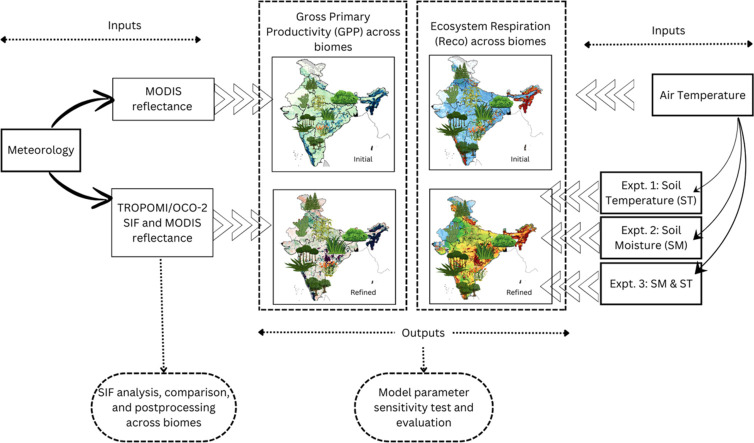

Specifications table

**Table:** 

Subject area:	Environmental Sciences
More specific subject area:	Terrestrial carbon flux estimation
Name of your method:	Refinement of LUE-based vegetation model
Name and reference of original method:	Mahadevan, P., Wofsy, S.C., Matross, D.M., Xiao, X., Dunn, A.L., Lin, J.C., Gerbig, C., Munger, J.W., Chow, V.Y., Gottlieb, E.W., 2008. A satellite-based biosphere parameterization for net ecosystem co2 exchange: Vegetation photosynthesis and respiration model (vprm). Global Biogeochemical Cycles 22. https://doi.org/10.1029/2006GB002735.
	Li, X., Xiao, J., 2019b. Mapping photosynthesis solely from solar-induced chlorophyll fluorescence: A global, fine-resolution dataset of gross primary production derived from oco-2. Remote Sensing 11, 2563. https://doi.org/10.3390/rs11212563. Luus, K.A., Lin, J.C., 2015. The polar vegetation photosynthesis and respiration model (polarvprm): A parsimonious, satellite data-driven model of high-latitude co2 exchange. Geoscientific Model Development Discussions 8, 979–1027. https://doi.org/10.5194/gmd-8–2655–2015, 2015. Gourdji, S.M., Karion, A., Lopez-Coto, I., Ghosh, S., Mueller, K.L., Zhou, Y., Williams, C.A., Baker, I.T., Haynes, K.D., Whetstone, J.R., 2022. A modified vegetation photosynthesis and respiration model (vprm) for the eastern usa and canada, evaluated with comparison to atmospheric observations and other biospheric models. Journal of Geophysical Research: Biogeosciences 127, e2021JG006290. https://doi.org/10.1029/2021JG006290.
Resource availability:	VPRM: https://doi.org/10.5281/zenodo.10245534 GOSIF: http://data.globalecology.unh.edu/ TROPOSIF: http://ftp.sron.nl/open-access-data-2/TROPOMI/tropomi/sif/v2.1/l2b/ ERA5: https://cds.climate.copernicus.eu/cdsapp#!/dataset/reanalysis-era5-land?tab=overview GLEAM: https://www.gleam.eu/#datasets

## Background

The terrestrial biosphere plays a significant role in sequestering a large fraction of CO_2_ from the atmosphere. Accurate quantification of these terrestrial carbon exchange fluxes between the atmosphere and biosphere is thus crucial for planning emission reduction measures at the regional scale. However, quantifying these fluxes for India poses challenges due to limited ground-based observations and the lack of sophisticated models capable of predicting these fluxes. In this study, we employed a light-use efficiency-based Vegetation Photosynthesis and Respiration Model (VPRM, Mahadevan et al. [24]) driven by the Moderate Resolution Imaging Spectroradiometer (MODIS) satellite data. The model provides high-resolution hourly fluxes of Gross Primary Productivity (GPP) and ecosystem Respiration (R_eco_) at a 0.1° × 0.1° grid cell. The VPRM model parameters to generate GPP and R_eco_ specific to the major vegetation classes over India are derived from MODIS reflectance such as Enhanced Vegetation Index (EVI) and Land Surface Water Index (LSWI), and modelled air temperature. The VPRM, previously validated in various other global regions [4,5,8], has demonstrated its potential in predicting vegetation carbon fluxes. To represent the vegetation fluxes of the region, the VPRM parameters must be calibrated using Eddy Covariance (EC) measurements covering major biomes, as was done in the previous studies [[3], [4], [5],^8^]. Given the inadequate availability of EC observations in India, this study opted for initial parameters calibrated against tropical biomes for the VPRM [3]. Further, we integrated additional observations currently accessible through satellite remote sensing platforms into the model in order to minimize the errors caused by the unavailability of ground-based EC measurements.

Solar-Induced Fluorescence (SIF) obtained through satellite retrievals can be used as a proxy for photosynthesis, as indicated by previous studies [28,34,38]. This study explores the potential of SIF to capture spatiotemporal features of vegetation productivity, thereby improving GPP predictions by the VPRM. To augment the model with observational input, we incorporated SIF retrievals from Orbiting Carbon Observatory-2 (OCO-2; Li and Xiao [18]) and Tropospheric Monitoring Instrument (TROPOMI; Guanter et al. [9]) (onboard Sentinel 5P), in addition to satellite-based information from the MODIS. The derived SIF-GPP relationship was then integrated into the VPRM GPP calculation, improving model performance as validated against EC data (see sections “Refinement of GPP estimates utilising SIF and Validation of refined models”).

Changes in precipitation patterns affect Soil Temperature (ST) and Soil Moisture (SM), influencing soil microbial activity and R_eco_ [1]. Previous studies show that SM and ST can affect R_eco_ rates, varying seasonally and spatially [31,34]. A simple linear relationship between air temperature and plant respiration as considered in the VPRM respiration equation [24] may be sufficient to represent ecosystem releases for regions where soil properties exert minimal influence on respiration dynamics. However, in the context of the Indian region, characterized by pronounced seasonal variations in precipitation patterns, it becomes imperative to account for the influence of soil properties on autotrophic and heterotrophic respiration [7,26,27]. Hence, in this study, we examine the influence of SM and ST content on R_eco_ and attempt to integrate these soil-related variables into the model's respiration equation (see section “Refinement of R_eco_ estimates”).

Overall, this article aims to demonstrate a data-driven approach providing a novel means of refining the GPP and R_eco_ estimations of flux distribution by VPRM for regions where ground-based flux measurements are the current limitation.

**Fig. 1:**
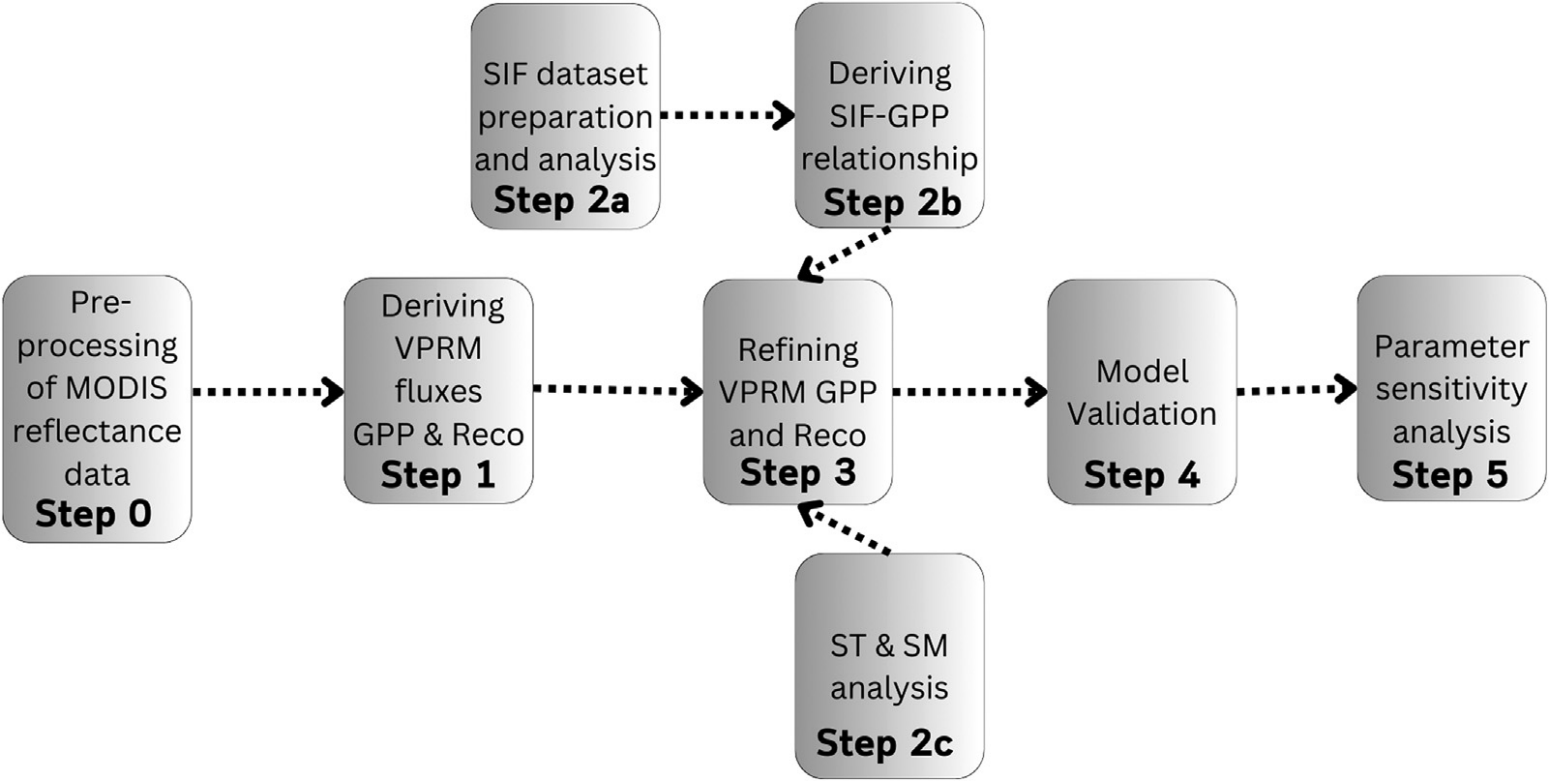
Flow chart showing the work flow.

Below, we describe the data and methods used in this paper. The flowchart in Fig. 1 provides an overview of the workflow.

## Method details

### MODIS-based estimations of biospheric CO_2_ fluxes

We used VPRM to derive estimates of GPP and R_eco_ for the Indian region. VPRM utilises the EVI and the LSWI, both derived from the remote sensing measurements collected by the MODIS on NASA's Terra and Aqua satellites, together with meteorology from ERA5 [12] to derive the VPRM GPP (GPP_VPRM_). We used the MODIS tiles of the surface reflectance dataset (MOD09A1) on sinusoidal grids at a 500 m spatial resolution with an 8-day interval to generate EVI and LSWI fields. Specifically, we used the red band (band 1), the near-infrared band (band 2), the blue band (band3) for deriving EVI, and the near-infrared band and the shortwave infrared band (band 6) for deriving LSWI. Air temperature information from ERA5 is used to generate VPRM R_eco_ fluxes (R_eco,VPRM_) taking into account the response of each vegetation class to temperature. For representing different biomes in VPRM, we used vegetation classification based on SYNMAP [15]. In the standard VPRM, GPP_VPRM_ and R_eco,VPRM_ are derived as follows:

(1) $$\text{GP}P_{\text{VPRM}}=\lambda \;\times P_{\text{scale}}\times T_{\text{scale}}\times W_{\text{scale}}\times \text{FPA}R_{\text{PAV}}\times SW_{\text{down}}\times \frac{1}{1+\frac{SW_{\text{down}}}{SW_{\text{down}0}}}$$

(2) $$R_{\text{eco},\text{VPRM}}=\alpha \;\times T_{\text{air}}+\beta$$

where λ is the light use efficiency term, and $\text{FPA}R_{\text{PAV}}$ is the fraction of incident radiation available for the photosynthetically active part of the vegetation. $\text{FPA}R_{\text{PAV}}$ is derived from MODIS EVI. $SW_{\text{down}}$ is prescribed from ERA5. $SW_{\text{down}0}$ is the half-saturation value. $T_{\text{scale}}$, $P_{\text{scale}}$, and $W_{\text{scale}}$ are dimensionless scalars representing the sensitivity of plants to changes in temperature, phenology, and water availability, respectively [2,24,37]. $T_{\text{scale}}$ is derived based on the equation developed for the terrestrial ecosystem model by Raich et al. [30] using ecosystem-specific temperature as follows:

(3) $$T_{\text{scale}}=\frac{\;\left(T_{\text{air}}-\;T_{\text{min}}\right)\;\left(T_{\text{air}}-T_{\text{max}}\right)}{\;\left(T_{\text{air}}-\;T_{\text{min}}\right)\;\left(T_{\text{air}}-T_{\text{max}}\right)-{\left(T_{\text{air}}-T_{\text{opt}}\right)}^{2}\;}$$

where $T_{\text{opt}}$, $T_{\text{max}}$, and $T_{\text{min}}$ represent optimal, maximum, and minimum temperatures for photosynthesis activity for each vegetation class. Photosynthesis is assumed to be absent above or below $T_{\text{max}}$ and $T_{\text{min}}$, respectively. $T_{\text{air}}$ is the hourly air temperature at 2 m prescribed from ERA5 [6]. In this study, we set $T_{\text{opt}}$, $T_{\text{max}}$, and $T_{\text{min}}$ to 20 °C, 45 °C, and 0 °C respectively. In Eq. (3), $T_{\text{air}}$ is constrained with a threshold value ($T_{\text{tshld}}$), and T_air_ below $T_{\text{tshld}}$ is set to $T_{\text{tshld}}$ for accounting for ecosystem respiration in winter times. $P_{\text{scale}}$ accounts for the effects of leaf age on photosynthesis; hence, it is set to 0 for water bodies and unclassified vegetation classes. $P_{\text{scale}}$ is assumed to always be 1 for the Evergreen vegetation class. For all vegetation classes other than Evergreen, we computed $P_{\text{scale}}$ as a function of LSWI except at the time of maximum greenness (representing full leaf expansion) as follows:

(4) $$P_{\text{scale}}=\frac{1+\text{LSWI}}{2}$$

For the maximum greenness time, $P_{\text{scale}}$ is set to 1. $W_{\text{scale}}$ is used to represent the effect of water stress on photosynthesis and is derived as follows:

(5) $$W_{\text{scale}}=\frac{1+\text{LSWI}}{1+\text{LSW}I_{\text{max}}}$$

where $\text{LSW}I_{\text{max}}$ is the maximum LSWI during the plant growing season per grid cell. The model parameters, specifically λ, $SW_{\text{down}0}$, α, and β are usually calibrated using EC measurements for each ecosystem.

This calibration process involves minimizing the least squares differences between the modelled fluxes and observations from eddy flux towers situated at discrete locations across major vegetation classes. This optimization approach, employed in studies such as Dayalu et al. [5] and Luus and Lin [22], is conducted to enhance the model's performance within the specified region.

Due to the limited availability of observational eddy flux measurements for calibration in the Indian region, we opted for VPRM model parameters (λ, $SW_{\text{down}0}$, α, and β) optimized based on the EC data from Amazonian Tropical biomes, as outlined in Table 1. However, it is important to note that these parameters may not accurately represent the subtropical biomes of India, potentially resulting in a decrease in model performance compared to simulations conducted in regions like Europe or North America. To decouple the influence of the initial model parameters, we also used another set of initial parameters that are optimized for European biomes (VPRM_EUR_) (refer to Table 1) in the model and examined their differences.

**Table 1:** List of VPRM initial parameters optimized against Amazonian Tropical and European biomes based on vegetation classes used in this study.

Vegetation class	Tropical parameters				European parameters			
	λ	$SW_{\text{down}0}$	α	β	λ	$SW_{\text{down}0}$	α	β
Grassland	0.13	157	0.026	0	−0.17	229.1	0.08	0.58
Cropland	0.12	646	0.004	0	−0.13	690.3	0.16	−0.01
Savanna	0.11	682	0.004	0	−0.11	682	0.004	0
Shrubland	0.08	303	0.02	0	−0.08	363	0.02	0
Deciduous forest	0.17	324	0.32	0	−0.19	271.4	0.14	0.82
Evergreen forest	0.21	501	0.16	0	−0.30	270.2	0.17	0.88
Mixed forest	0.25	206	0.34	0	−0.28	236.6	0.22	0.43

*Units are as follows: λ: µmol CO_2_ m^−2^ s^−1^/ µmol $SW_{\text{down}}$ m^−2^ s^−1^; α: µmol CO_2_ m^−2^ s^−1^/ °C; β: µmol CO_2_ m^−2^ s^−1^; $SW_{\text{down}0}$: µmol CO_2_ m^−2^ s^−1^.

## Satellite-based SIF observations across Indian biomes

We hypothesize that utilising the satellite remote sensing measurements of SIF may partly address the challenge posed by the limited availability of adequate EC observations for calibrating the VPRM parameter across India. SIF is the signal emitted by the chlorophyll pigment in plants upon the absorption of sunlight during photosynthesis. Hence, SIF is considered as a proxy for photosynthesis [28,34,38]. To improve the GPP distribution in VPRM two recently available SIF products are employed. We utilised the global 0.1° × 0.1° gridded Level 2B product from TROPOMI, denoted as TROPOSIF, focusing on the 743–748 nm fitting window. Daily SIF data obtained from the TROPOMI onboard the Sentinel-5P satellite are available (http://ftp.sron.nl/open-access-data-2/TROPOMI/tropomi/sif/v2.1/l2b/). Additionally, we employed another SIF product called GOSIF_v2 (http://data.globalecology.unh.edu/; Li and Xiao [19]) derived from observations made by the OCO-2 using a machine learning approach. GOSIF_v2 (hereafter referred to as GOSIF) is available at a spatial resolution of 0.05° and a temporal scale of 8 days. GOSIF retrievals at 757 nm are used here. Based on the availability of the data, GOSIF data from 2016 to 2020 and TROPOSIF data from May 2018 to 2020 are employed in this study. Daily TROPOSIF data is aggregated on an 8-day scale to match the temporal resolution of GOSIF.

### Minimizing scale mismatch between GOSIF and TROPOSIF

**Fig. 2:**
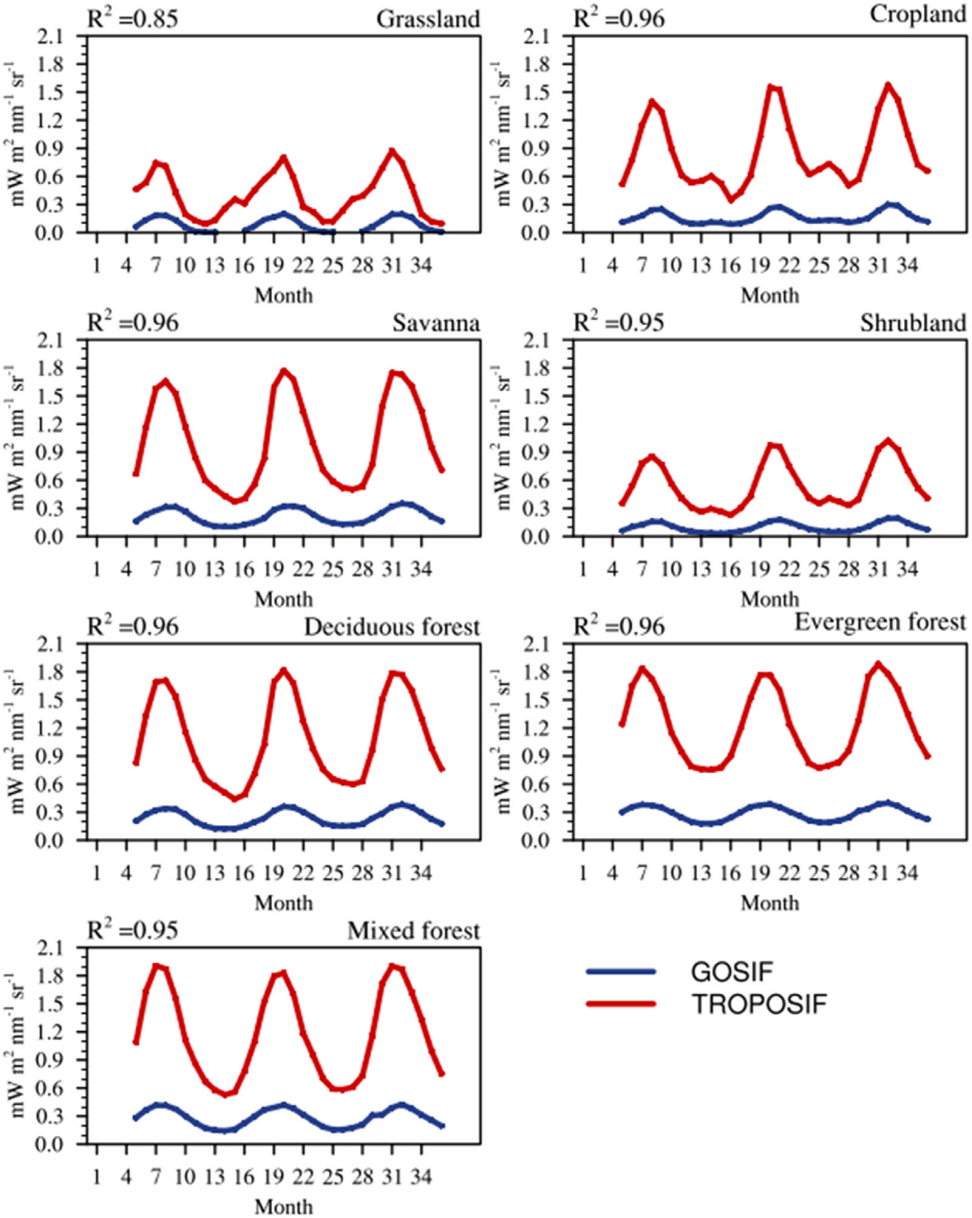
Time series of monthly averaged SIF (GOSIF and TROPOSIF) across different biomes over India from 2018 to 2020. The vegetation classification based on SYNMAP is used to represent SIF for different biomes. (For interpretation of the references to color in this figure legend, the reader is referred to the web version of this article.)

We made the biome-specific analyses of SIF products, deducing their spatial and temporal characteristics over Indian biomes from 2018 to 2020 (period selection is based on common data availability). For the spatial analysis, GOSIF data have been re-gridded to 0.1° × 0.1° to match the resolution of TROPOSIF. The 8-day averaged SIF products from GOSIF and TROPOSIF reasonably agree with each other across biomes with R^2^ ranging from 0.45 to 0.62 except for Grassland (R^2^ = 0.22) (see Table 2). A similar good agreement between SIF retrievals from OCO-2 and TROPOMI on a global scale is also reported by K¨ohler et al. [17] and Guanter et al. [9]. Fig. 2 illustrates the comparison between the monthly averaged TROPOSIF and GOSIF across various vegetation classes of India spanning from 2018 to 2020. The vegetation classification is made according to the SYNMAP. A robust correlation exists between TROPOSIF and GOSIF products (R^2^ > 0.85). Overall, we find that TROPOSIF values are ∼4 times greater than GOSIF over the study region for all the biomes except for Grassland, where the biome-specific TROPOSIF is ∼3 times larger than GOSIF. A scaling factor separately for each vegetation class (S_GOSIF,vg_) is applied to minimize these differences as follows:

(6) $$\text{TROPOSI}F_{\text{vg}\;}=S_{\text{GOSIF},\text{vg}}\times \text{GOSI}F_{\text{vg}}$$

where S_GOSIF,vg_ is the scaling factor representing the factorial difference between TROPOSIF and GOSIF based on vegetation type, vg. Table 2 provides S_GOSIF,vg_ values used across biomes. A similar upscaling of OCO-2 SIF is also done by K¨ohler et al. [17] and Guanter et al. [9] to compare the fields with TROPOSIF on a global scale.

**Table 2:** List of scalars applied to GOSIF (based on SIF retrievals at 757 nm) on an 8-day time step, specific to each vegetation type. The squared correlation coefficients (R^2^) between GOSIF and TROPOSIF from 2018 to 2020 across different vegetation classes are indicated. R^2^ in the annual GPP from VPRM with annual GOSIF and TROPOSIF across different vegetation classes are also provided for the year 2019.

Vegetation class	S_GOSIF_	R^2^ (GOSIF vs TROPOSIF)	R^2^ (GPP_VPRM_ vs TROPOSIF)	R^2^ (GPP_VPRM_ vs GOSIF)
Grassland	2.81	0.22	0.22	0.52
Cropland	4.62	0.45	0.48	0.53
Savanna	4.35	0.56	0.22	0.29
Shrubland	4.35	0.62	0.77	0.84
Deciduous forest	4.17	0.56	0.46	0.55
Evergreen forest	4.02	0.52	0.59	0.59
Mixed forest	3.94	0.55	0.44	0.52

### Spatio-temporal patterns of SIF over Indian biomes

**Fig. 3:**
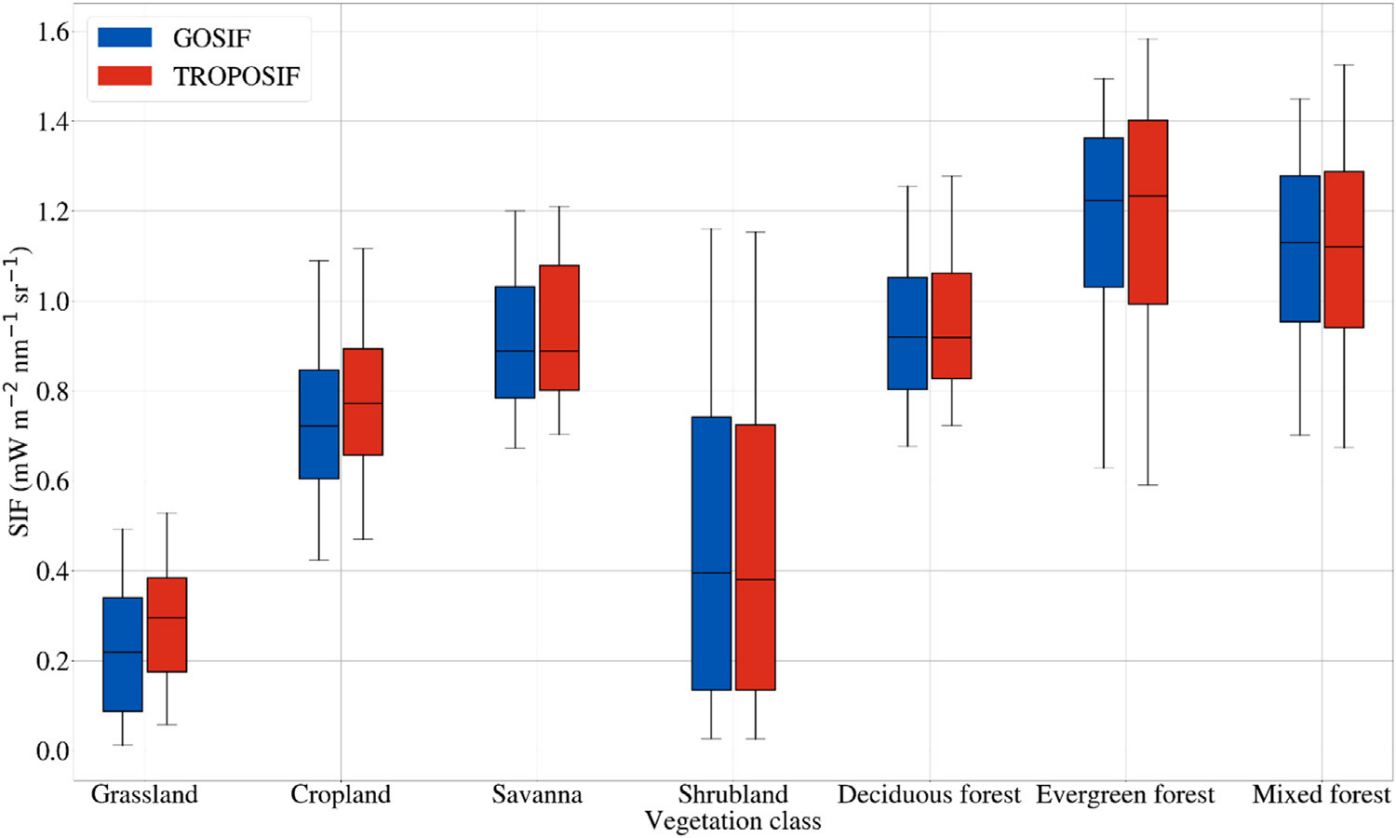
Comparison between annual SIF retrievals from OCO-2 (GOSIF) and TROPOSIF across vegetation classes over India averaged for 2019 and 2020. GOSIF (estimated at 757 nm) is scaled by respective biome-specific scaling factors (see Table 2) to compare with TROPOMI SIF (estimated at 743 nm and 748 nm). The upper and lower limit of the box shows the 5^th^ and 95^th^ percentile of the data and the center line shows the median. All the values that are 1.5 times higher than the 5^th^ and 95^th^ percentile are considered outliers and are removed from the graph. (For interpretation of the references to color in this figure legend, the reader is referred to the web version of this article.)

Fig. 3 compares scaled GOSIF and TROPOSIF across different biomes. Annually, the highest SIF values (GOSIF, mean/min/max: 1.16/0.62/1.49 mW m^−2^ sr^−1^ nm^−1^ and TROPOSIF, mean/min/max: 1.17/0.59/1.58 mW m^−2^ sr^−1^ nm^−1^) are exhibited by Evergreen forest, and the lowest values are observed (GOSIF, mean/min/max: 0.22/0.011/0.49 mW m^−2^ sr^−1^ nm^−1^, TROPOSIF, mean/min/max: 0.28/0.05/0.53 mW m^−2^ sr^−1^ nm^−1^) over Grassland vegetation. The above values are based on the annual average across different vegetation types for 2019 and 2020 per grid. On an annual scale, large spatial variability in the SIF values is exhibited by Shrublands and the least by Deciduous forest and Grasslands. Compared to 2019, SIF values from GOSIF of the year 2020 for Cropland, Savanna, Shrubland, Deciduous forest, and Evergreen forest show enhancement in the range of 0.01 mW m^−2^ sr^−1^ nm^−^1 to 0.23 mW m^−2^ sr^−1^ nm^−1^, with Grassland showing no enhancement. Mixed forest biomes exhibited a decline in SIF value (−0.005 mW m^−2^ sr^−1^ nm^−1^) in 2020 compared to the previous year. Like GOSIF, TROPOSIF also indicates no increments in SIF values for Grasslands, while other ecosystems show an annual augmentation between 0.04 mW m^−2^ sr^−1^ nm^−1^ to 0.11 mW m^−2^ sr^−1^ nm^−1^.

We further analysed SIF variability against GPP from VPRM. While both GOSIF (upscaled) and TROPOSIF products are in good agreement with GPP_VPRM_ over most of the vegetation classes in our study (e.g., R^2^ = 0.77 to 0.84 for Shrubland), we found a weak correlation between SIFs and standard VPRM-derived GPP for Savanna (R^2^ = 0.22 to 0.29). The above correlation values are based on the annually averaged data analysis from 2019 (see Table 2). It is noteworthy that the SIF-GPP relationship can become weak in certain environmental conditions such as drought (e.g., Shekhar et al. [33]) and be variable within certain biomes based on leaf physiology (e.g., Wu et al. [36]). However, a future study is needed to elucidate SIF-GPP relationships in India across different biomes in drought and wet conditions.

## Deriving SIF-GPP relationship across biomes

We followed Li and Xiao [19] to establish the relationship between SIF and GPP across Indian biomes. Here also we assume that the GPP varies linearly with SIF [35,39]. Previous studies have used different approaches to derive GPP from SIF, which also varied across biomes. Some studies have used a linear relationship between SIF and GPP, while others have explored non-linear relationships [11,20,39]. Additionally, there are studies that have considered universal relationships for all vegetation types versus those specific to biomes [10,35]. Besides vegetation type, physiological as well as environmental factors such as temperature, moisture, and radiation also significantly influence the SIF-GPP relationship [4,21,23,29]. At the leaf level, the relationship resembles a typical light response curve of a leaf, where photosynthesis reaches saturation at moderate light intensity while SIF continues to increase proportionally with light intensity [25]. This calls for future studies focusing on elucidating the relationship between SIF and GPP taking into account the environmental, physiological, and geographical factors specific to the Indian region. The above efforts require observational datasets of both SIF and GPP across various biomes at the field level representing different environmental conditions, which are currently limited over India. Because of the data limitation, the present study does not specifically derive the uncertainties in the SIF-GPP relationship though stringent quality filtering is applied to the SIF measurements used. Our approach assumes the above uncertainty to be negligible relative to other major model errors of GPP estimation. However, using SIF alone is insufficient for accurate GPP estimation due to the discrepancies in the quantitative relationships as explained above. Hence, the present study integrates SIF and auxiliary environmental information together with the surface reflectance data to minimize uncertainties in GPP estimation*.*

Here, the 8-day averaged GPP from GOSIF (denoted as GPP_GOSIF_) is derived as follows:

(7) $$\text{GP}P_{\text{GOSIF}}\left(\text{vg}\right)=\gamma_{\text{GOSIF},\text{vg}}\times \;\text{GOSIF}\left(\text{vg}\right)+C_{\text{GOSIF},\text{vg}}$$

where $\gamma_{\text{vg}}$ is the factor converting GOSIF to GPP_GOSIF_ for each vegetation class. Similarly, $C_{\text{GOSIF},\text{vg}}$ represents the constant specific to each vegetation class, vg. Our derived scalars for converting SIF to GPP are different from Li and Xiao [19] due to the differences in Indian biomes, their classifications, and the upscaling of the GOSIF product (see Table 3). We utilised the above SIF-GPP relationship to derive GPP from TROPOSIF. When using SIF products from TROPOSIF, the factor of difference between GOSIF and TROPOSIF values (S_GOSIF,vg_, see Table 2) must be taken into account as follows:

(8) $$\text{GP}P_{\text{TROPOSIF}}\left(\text{vg}\right)=\;\gamma_{\text{TROPOSIF},\text{vg}}\times \;\text{TROPOSIF}\left(\text{vg}\right)+C_{\text{TROPOSIF},\text{vg}}$$

where

(9) $$\gamma_{\text{TROPOSIF},\text{vg}}=\frac{\gamma_{\text{GOSIF},\text{vg}}}{S_{\text{GOSIF},\text{vg}}}$$

(10) $$C_{\text{TROPOSIF},\text{vg}}=\frac{C_{\text{GOSIF},\text{vg}}}{S_{\text{GOSIF},\text{vg}}}$$

**Table 3:** Biome-specific scalars used for the conversion of TROPOSIF to GPP_TROPOSIF_ and GOSIF to GPP_GOSIF_ across different vegetation classes across India (see section “Deriving SIF-GPP relationship across biomes”).

Vegetation class	$\gamma_{\text{TROPOSIF}}$ (mW m^−2^ sr^−1^ nm^−1^)/ (µmol m^−2^ s^−1^)	C_TROPOSIF_	$\gamma_{\text{GOSIF}}$ (mW m^−2^ sr^−1^ nm^−1^)/ (µmol m^−2^ s^−1^)	C_GOSIF_
Grassland	7.84	0.40	22.03	1.12
Cropland	4.81	0.22	22.22	1.01
Savanna	5.12	0.32	22.27	1.39
Shrubland	5	0.39	21.75	1.69
Deciduous forest	5.35	0.34	22.30	1.41
Evergreen forest	5.47	0.64	21.98	2.57
Mixed forest	5.59	0.61	22.02	2.40

**Fig. 4:**
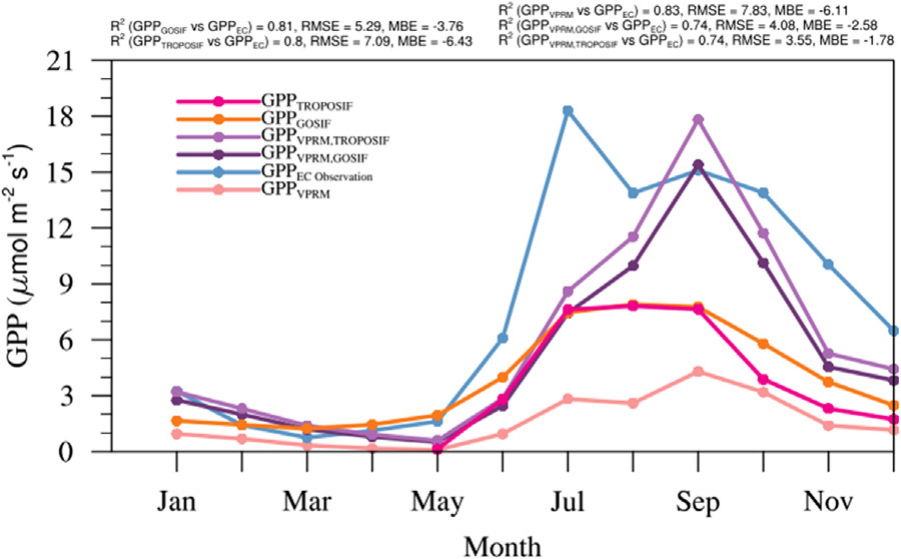
Comparison of monthly averaged GPP from EC observations with GPP_GOSIF_, GPP_TROPOSIF_, GPP_VPRM_, GPP_VPRM,GOSIF_, and GPP_VPRM,TROPOSIF_ for Betul during 2018. Note that TROPOSIF data has only been available since May 2018. (For interpretation of the references to color in this figure legend, the reader is referred to the web version of this article.)

Table 3 provides the scalars used for converting TROPOSIF to GPP_TROPOSIF_. To evaluate how well these GPP products (GPP_VPRM_, GPP_TROPOSIF_ and GPP_GOSIF_) capture the observed variability, we validated them using EC observations obtained from the Betul (21°51′46.84″ N latitude and 77°25′33.67″E longitude, Madhya Pradesh; Jha et al. [13]) site in Central India. The tower is situated in a homogeneous mixed Deciduous forest with a tropical climate, has been operational since November 2011, and is located at 507 m above sea level. Additional information about the site, instrumentation details, and data preprocessing at Betul can be found in [13,32]. Here, the utilization of EC observations is restricted to just one site, primarily because of constraints related to data availability. GPP_VPRM_ captured the seasonal pattern (R^2^ = 0.83) better than SIF-based GPP products (GPP_TROPOSIF_ and GPP_GOSIF_) but with a larger model-observation bias (see Fig. 4). While these results demonstrate the potential of the VPRM in predicting temporal variations of observed GPP, it indicates the need for further calibration of model parameters. As a result, we have chosen to integrate SIF observational information to calibrate the VPRM GPP parameters. This strategic calibration is expected to reproduce the observed variations in GPP.

## Refinement of GPP estimates utilising SIF

We integrated 8-day averaged SIF observations to modify the $\text{GP}P_{\text{VPRM}}$, as follows:

(11) $$\text{GP}P_{\text{VPRM},\text{SIF}}\left(i,j,t,\text{vg}\right)=\eta_{\text{vg}}\times \text{GP}P_{\text{VPRM}}\left(i,j,t,\text{vg}\right)+\varepsilon_{\text{vg}}$$

Here, $\text{GP}P_{\text{VPRM},\text{SIF}}$ refers to either GPP refined based on GOSIF (further denoted as $\text{GP}P_{\text{VPRM},\text{GOSIF}}$) or GPP refined based on TROPOSIF (further denoted as $\text{GP}P_{\text{VPRM},\text{TROPOSIF}}$). i, j, and t represent latitude, longitude, and time coordinates, respectively. $\eta_{\text{vg}}$ is the scaling factor corresponding to the specific vegetation class, applied to GPP_VPRM_ to include the information provided by SIF (see Table 4). $\eta_{\text{vg}}$ is thus:

(12) $$\eta_{\text{vg}}=\frac{\sum_{i=1}^{n1}\sum_{j=1}^{n2}\sum_{t=1}^{n3}\left(\text{GP}P_{\text{SIF}}\left(i,j,t,\text{vg}\right)\;\times \text{GP}P_{\text{VPRM}}\left(i,j,t,\text{vg}\right)\right)}{\sum_{i=1}^{n1}\sum_{j=1}^{n2}\sum_{t=1}^{n3}\text{GP}P_{\text{VPRM}}\left(i,j,t,\text{vg}\right)}$$

$\varepsilon_{\text{vg}}$ in Eq. (11) represents the vegetation-specific error term or the y-intercept between $\text{GP}P_{\text{SIF}}\left(\text{vg}\right)$ and $\text{GP}P_{\text{VPRM}}\left(\text{vg}\right)$. n1, n2, and n3 represent the number of latitude, longitude, and time indices per vegetation class. The refinement improved model performance with modified models ($\text{GP}P_{\text{VPRM},\text{GOSIF}}$ and $\text{GP}P_{\text{VPRM},\text{TROPOSIF}}$) showing a reduction in bias and spread from the observation (see Fig. 4). It is also worth mentioning that the accuracy of GPP calculated using the demonstrated approach relies on satellite SIF and reflectance products and is expected to improve with dedicated satellite instruments designed for these measurements at high spatial and temporal resolutions.

**Table 4:** Biome-specific scalars used for creating GPP_VPRM,GOSIF_ and GPP_VPRM,TROPOSIF_.

Vegetation class	η	
	TROPOSIF	GOSIF
Grassland	3.2	3.3
Cropland	1.6	1.7
Savanna	3.7	2.3
Shrubland	3.3	2.2
Deciduous forest	2.4	1.7
Evergreen forest	1.7	1.2
Mixed forest	2.3	1.5

## Refinement of R_eco_ estimates

**Fig. 5:**
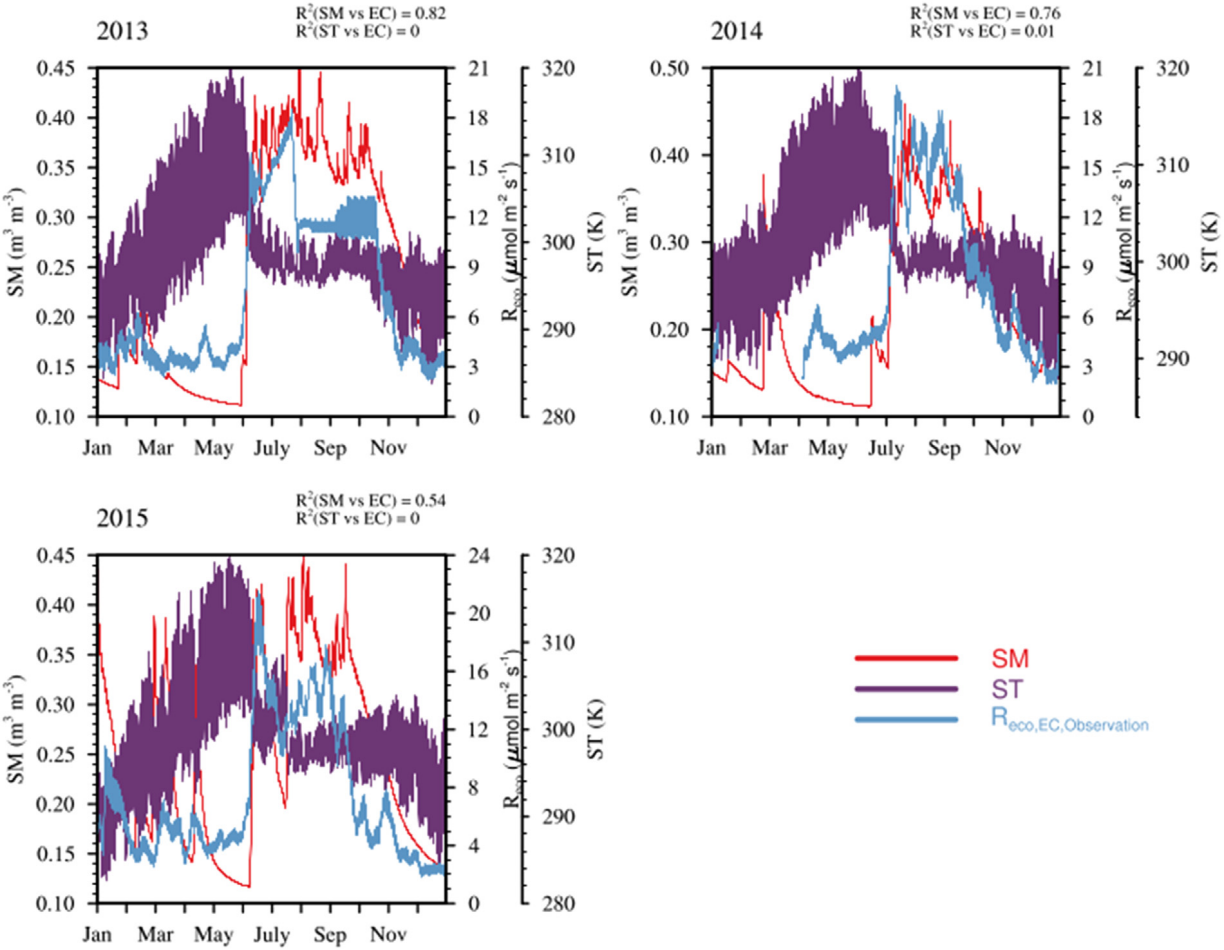
Temporal variation (3 hourly) in the R_eco_ fluxes compared against soil moisture and soil temperature fields from HRLDAS for the Betul site. (For interpretation of the references to color in this figure legend, the reader is referred to the web version of this article.)

The influence of soil properties may become crucial in influencing both autotrophic and heterotrophic respiration, particularly in regions with distinct wet and dry seasons [7,26,27]. To examine whether SM and ST influence R_eco_ in the Indian region, we analysed the correlation between R_eco_ data from the Betul EC site and surface SM and ST fields obtained from the high-resolution land data assimilation system (HRLDAS; Chen et al., 2007) based on the Noah land surface model (LSM). The HRLDAS provides 3-hourly SM and ST fields at a spatial resolution of 4 km from 2012 to 2017. We compared the SM and ST fields from HRLDAS with EC fluxes by utilising the nearest grid point to the Betul site location. For this comparison, the hourly fluxes from Betul are averaged to 3-hourly (see Fig. 5). A strong dependence of R_eco_ on SM was found (R^2^ > 0.5), but ST showed minimal effect on the observed respiration flux pattern. We also use the surface SM fields from GLEAM v3 (spatial resolution: 0.25° × 0.25°, temporal resolution: daily) (https://www.gleam.eu/#datasets; Martens et al. [25]) model and level 2 (7 - 28 cm) ST from ERA5 (spatial resolution: 0.1° × 0.1°, temporal resolution: hourly) (https://cds.climate.copernicus.eu/cdsapp#!/dataset/reanalysis-era5-land?tab=overview; Hersbach et al. [12]) reanalysis product as they provide latest data with more temporal coverage.

We designed three experiments for improving predictions of R_eco_ by integrating the corresponding equation with 1) SM only, 2) ST only, and 3) both ST and SM. Additionally, we used global data products FLUXNET-Random Forest (https://db.cger.nies.go.jp/DL/10.17595/20200227.001.html.en, Jiye [14]) and FLUXCOM (https://www.bgc-jena.mpg.de/geodb/projects/DataDnld.php, Jung et al. [16]) generated from a network of flux towers worldwide for adjusting the magnitude of resulted fluxes for each vegetation classes. The FLUXNET-Random forest product is generated by upscaling EC observations from FLUXNET 2015 using the Random Forest method, resulting in a global gridded dataset. This approach incorporates satellite and meteorological data. Conversely, FLUXCOM utilises machine learning techniques to integrate energy flux measurements from FLUXNET towers with remote sensing and meteorological data to estimate carbon fluxes on a global grid. Even though both products rely on the same set of observations, the accuracy of carbon flux estimates is influenced by factors such as the choice of machine learning algorithm, predictor variables, input data, and the representation of various ecosystems. The FLUXNET-Random forest product is referred to as FLUXNET for the rest of the study. The upscaling process involves separate utilization of FLUXCOM and FLUXNET for comparative analysis purposes. The scaling is done separately for each vegetation class by considering respiration fluxes corresponding to each vegetation class in our domain.

The respiration equation is modified as follows:

Based on experiment 1:

(13) $$R_{\text{eco},\text{VPRM},\text{SM}}\left(i,j,\text{vg}\right)=\nu_{\text{vg},\text{SM}}\times \;\text{SM}\left(i,j,\text{vg}\right)+\kappa_{\text{vg},\text{SM}}\times \;\left(\alpha_{\text{vg}}\times T_{\text{air}}\left(i,j,\text{vg}\right)+\beta_{\text{vg}}\right)$$

Based on experiment 2:

(14) $$R_{\text{eco},\text{VPRM},\text{ST}}\left(i,j,\text{vg}\right)=\tau_{\text{vg},\text{ST}}\times \;\text{ST}\left(i,j,\text{vg}\right)+\kappa_{\text{vg},\text{ST}}\times \;\left(\alpha_{\text{vg}}\times T_{\text{air}}\left(i,j,\text{vg}\right)+\beta_{\text{vg}}\right)$$

Based on experiment 3:

(15) $$R_{\text{eco},\text{VPRM},\text{SMST}}\left(i,j,\text{vg}\right)=\tau_{\text{vg}}\times \;\text{ST}\left(i,j,\text{vg}\right)+\nu_{\text{vg}}\times \;\text{SM}\left(i,j,\text{vg}\right)+\kappa_{\text{vg}}\times \;\left(\alpha_{\text{vg}}\times T_{\text{air}}\left(i,j,\text{vg}\right)+\beta_{\text{vg}}\right)$$

where, $\nu_{\text{vg},\text{SM}}$, $\kappa_{\text{vg},\text{SM}}$, $\tau_{\text{vg},\text{ST}}$, $\kappa_{\text{vg},\text{ST}}$, $\tau_{\text{vg}}$, $\nu_{\text{vg}}$, and $k_{\text{vg}}$ represent the vegetation specific parameters, adjusted against observation-based respiration fluxes (FLUXNET or FLUXCOM).

**Fig. 6:**
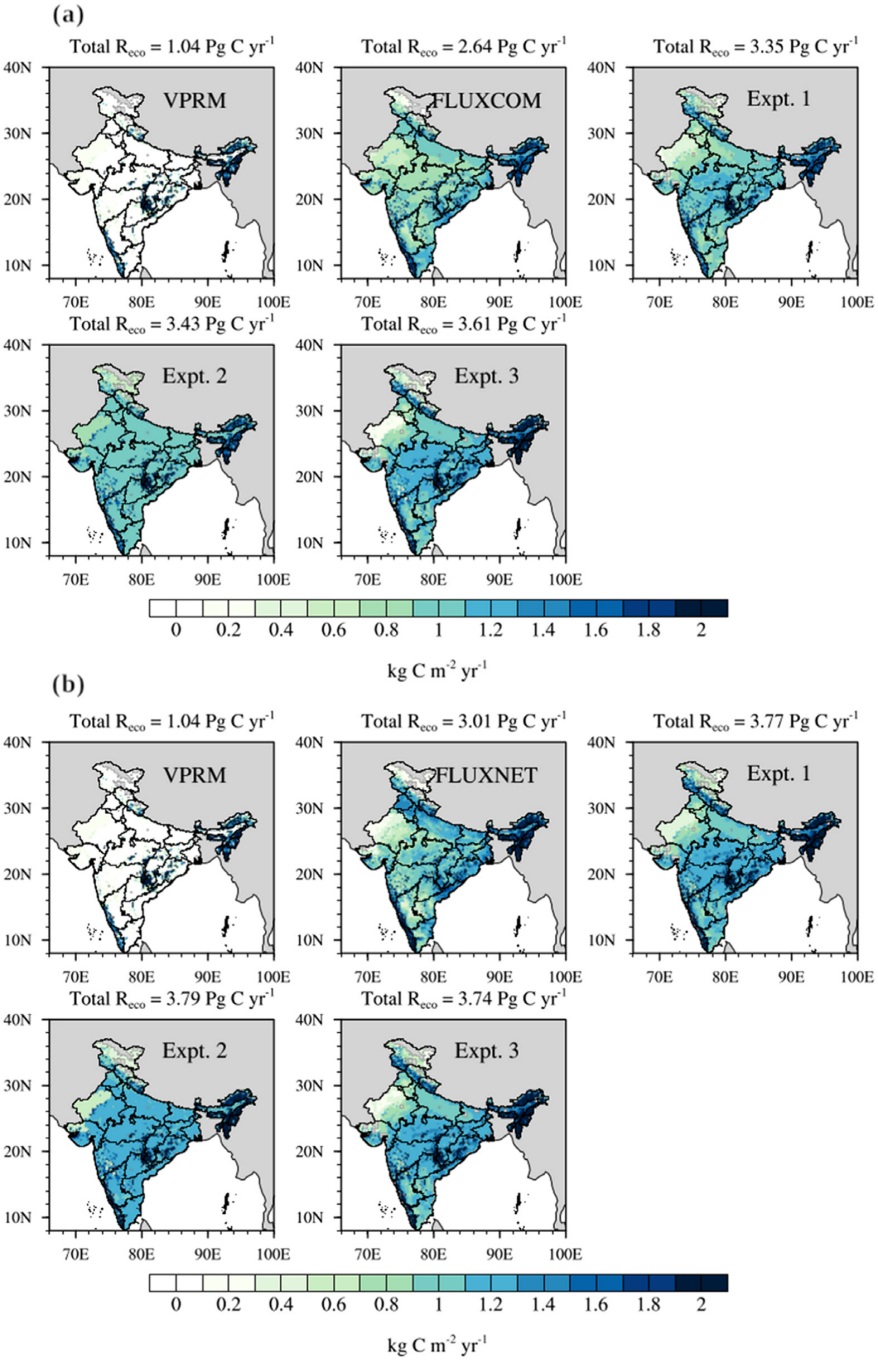
Comparison of the annual distribution of R_eco_ fluxes from standard and refined VPRM for 2016. Shows a) the refined VPRM simulations (Expt. 1, Expt. 2, and Expt. 3) in which κ-related parameters for each vegetation class are adjusted with FLUXCOM dataset, and b) the same as (a), but κ-related parameters for each vegetation class are adjusted with FLUXNET dataset. (For interpretation of the references to color in this figure legend, the reader is referred to the web version of this article.)

Table 5 provides the list of vegetation-specific scalars used for R_eco,VPRM_ refinements using ST fields from ERA5 and SM fields from GLEAM. Fig. 6 shows the distribution of modified R_eco_ fluxes based on standard VPRM, experiments 1, 2, and 3, and estimates from FLUXCOM and FLUXNET data.

**Table 5:** List of VPRM standard and refined respiration parameters based on vegetation classes adjusted using FLUXNET.

Vegetation class	$\nu_{\text{vg},\text{SM}}$	$\kappa_{\text{vg},\text{SM}}$	$\tau_{\text{vg},\text{ST}}$	$\kappa_{\text{vg},\text{ST}}$	$\tau_{\text{vg}}$	$\nu_{\text{vg}}$	$\kappa_{\text{vg}}$
Grassland	1545.8	3.90	0.002	3.90	−0.0023	2790.4	3.96
Cropland	7997.9	0.20	0.009	0.40	−0.0008	8588.3	0.20
Savanna	9770.4	−0.10	0.01	−0.09	−0.0009	10,321.2	−0.07
Shrubland	4390.4	0.60	0.003	0.90	−0.001	5059.4	0.72
Deciduous forest	10,059.1	−0.03	0.01	0.01	−0.003	11,684.6	0.02
Evergreen forest	7147.7	0.50	0.01	0.40	0.005	4505.6	0.44
Mixed forest	7488.6	0.30	0.01	0.40	−0.005	10,214.6	0.30

*Units are as follows: τ: µmol CO_2_ m^−2^ s^−1^ K^−1^; ν: µmol CO_2_ m^−2^ s^−1^ m^−3^ m^3^; κ: dimensionless.

## Validation of refined models

**Fig. 7:**
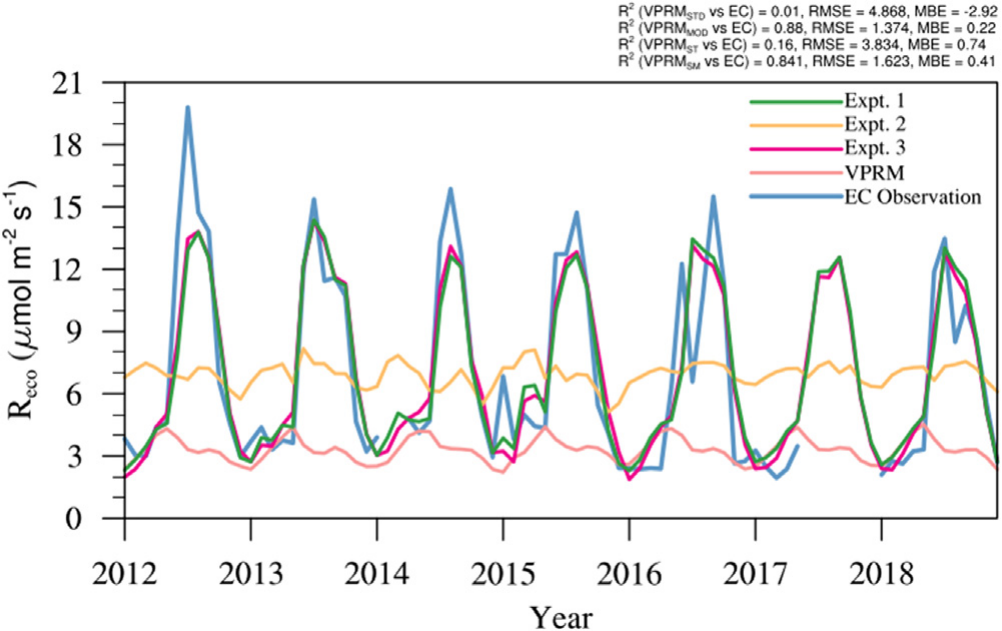
Comparison of monthly averaged EC observations with R_eco_ simulations over Betul for the period 2012 to 2018. (For interpretation of the references to color in this figure legend, the reader is referred to the web version of this article.)

The validation of refined GPP and R_eco_ models was done by comparing them with EC observation from Betul site (see Fig. 4, Fig. 7). The intercomparison of GPP_VPRM,GOSIF_ and GPP_VPRM,TROPOSIF_ with the standard GPP_VPRM_ shows remarkable improvement in the model performance with a significant reduction in RMSE (RMSE: VPRM = 7.83 µmol m^−2^ s^−1^, µmol m^−2^ s^−1^ VPRM_GOSIF_ = 4.9 µmol m^−2^ s^−1^, and VPRM_TROPOSIF_ = 4.3 µmol m^−2^ s^−1^) and MBE (MBE: VPRM = −6.11 µmol m^−2^ s^−1^, VPRM_GOSIF_ = −3.3 µmol m^−2^ s^−1^, VPRM_TROPOSIF_ = −2.6 µmol m^−2^ s^−1^) values. The above levels of model improvements confirm the potential of using high-resolution satellite-derived SIF in capturing the seasonal cycle of GPP at an ecosystem level. VPRM respiration modified using SM (Experiment 1: R^2^ = 0.80) shows much improvement in model prediction than when ST (Experiment 2: R^2^ = 0.06) alone is used (see Fig. 7). VPRM respiration modified using both SM and ST (Experiment 3) shows slightly better improvement than using only SM (see Table 6). The model-observation bias reduced considerably, with RMSE reducing from 5.7 µmol m^−2^ s^−1^ to 1.9 µmol m^−2^ s^−1^ and MBE reducing from −3.5 µmol m^−2^ s^−1^ to −0.01 µmol m^−2^ s^−1^ when both SM and ST are added to VPRM respiration. In general, incorporating the SM and ST in addition to air temperature in the R_eco_ calculation in the VPRM improves the model's ability to produce more realistic values over the Deciduous ecosystem of Betul.

**Table 6:** Comparison of monthly averaged R_eco_ fluxes from VPRM model simulations against EC observations for Betul from 2012 to 2018.

Variable name	R^2^	RMSE (µmol m^−2^ s^−1^)	MBE (µmol m^−2^ s^−1^)
R_eco,VPRM_	0.02	5.7	−3.50
R_eco,VPRM,ST_	0.06	4.4	0.08
R_eco,VPRM,SM_	0.80	2.0	−0.01
R_eco,VPRM,SMST_	0.82	1.9	−0.01

## VPRM initial parameter sensitivity test

**Fig. 8:**
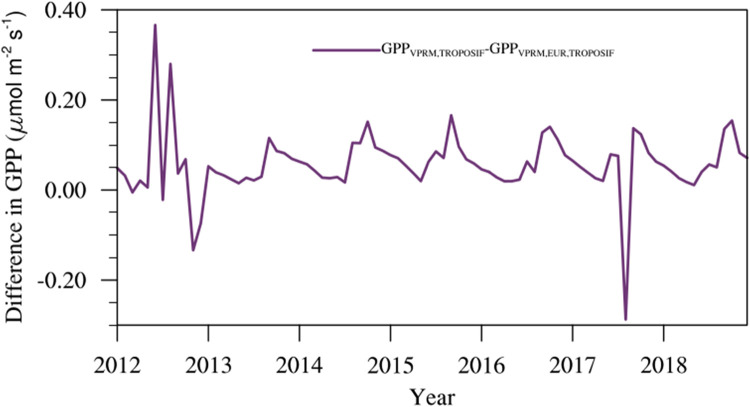
The difference in GPP simulations among the refined VPRM runs using the Amazonian Tropical and European parameters for the Betul site.

As we cannot specifically calibrate the VPRM model parameters for the Indian region using EC observations, assessing the sensitivity of refined model simulations to the chosen initial VPRM model parameters becomes important. For this, we performed the standard VPRM model simulations of GPP using model parameters calibrated for European biomes (denoted as GPP_VPRM,EUR_). We then refined GPP_VPRM,EUR_ using TROPOSIF to generate GPP_VPRM,EUR,TROPOSIF_. The refinement is done following the same approach applied in GPP_VPRM_ to generate GPP_VPRM,TROPOSIF_ as detailed in section “Refinement of GPP estimates utilising SIF”. Fig. 8 shows the difference between GPP_VPRM,TROPOSIF_ and GPP_VPRM,EUR,TROPOSIF_ for Betul observation site, giving an average GPP difference within 0.05 µmol m^−2^ s^−1^ i.e. the choice of initial model parameters in VPRM does not significantly impact the model refinements that receive additional observational information from SIF.

## Summary

This research presents a methodology to improve the LUE-based model that harnesses the capabilities of satellite-derived SIF data within vegetation models to produce reliable GPP estimates. Our approach holds significant promise for regions where EC observational data are scarce. The validation of the refined vegetation model against EC observations from the Betul site indicates that integrating SIF has led to improved model performance. This enhancement has resulted in model values closely aligning with the observed data. Furthermore, we observe that the TROPOMI-based product exhibits superior performance in predicting variations in observed GPP compared to the OCO-2-based product. We showed the importance of incorporating soil-related information in the VPRM respiration equation. Our innovative approach of including ST and SM in the model respiration in addition to air temperature significantly improved the model's ability to reproduce the observed respiration fluxes. The sensitivity test conducted to examine the impact of initial model parameters on the refined model indicates a negligible influence of the initial parameters on the ultimate output.

## Limitations

Not applicable

## Ethics statements

Not applicable

## CRediT authorship contribution statement

**Aparnna Ravi:** Data curation, Formal analysis, Investigation, Validation, Visualization, Writing – original draft. **Dhanyalekshmi Pillai:** Conceptualization, Funding acquisition, Methodology, Supervision, Writing – review & editing. **Vishnu Thilakan:** Writing – review & editing. **Thara Anna Mathew:** Visualization.

## Declaration of competing interest

The authors declare that they have no known competing financial interests or personal relationships that could have appeared to influence the work reported in this paper.

## Data Availability

Data will be made available on request.
